# Dynamic Evaluation of the Degradation Process of Vibration Performance for Machine Tool Spindle Bearings

**DOI:** 10.3390/s23115325

**Published:** 2023-06-04

**Authors:** Liang Ye, Wenhu Zhang, Yongcun Cui, Sier Deng

**Affiliations:** 1 School of Mechatronics Engineering, Henan University of Science and Technology, Luoyang 471003, China; 9945070@haust.edu.cn (L.Y.); zwh@haust.edu.cn (W.Z.); 9906172@haust.edu.cn (Y.C.); 2 National United Engineering Laboratory for Advanced Bearing Tribology, Henan University of Science and Technology, Luoyang 471023, China

**Keywords:** machine tool spindle bearings, degradation probability, vibration performance maintaining reliability, uncertainty, optimal performance state

## Abstract

Real-time condition monitoring and fault diagnosis of spindle bearings are critical to the normal operation of the matching machine tool. In this work, considering the interference of random factors, the uncertainty of the vibration performance maintaining reliability (VPMR) is introduced for machine tool spindle bearings (MTSB). The maximum entropy method and Poisson counting principle are combined to solve the variation probability, so as to accurately characterize the degradation process of the optimal vibration performance state (OVPS) for MTSB. The dynamic mean uncertainty calculated using the least-squares method by polynomial fitting, fused into the grey bootstrap maximum entropy method, is utilized to evaluate the random fluctuation state of OVPS. Then, the VPMR is calculated, which is used to dynamically evaluate the failure degree of accuracy for MTSB. The results show that the maximum relative errors between the estimated true value and the actual value of the VPMR are 6.55% and 9.91%, and appropriate remedial measures should be taken before 6773 min and 5134 min for the MTSB in Case 1 and Case 2, respectively, so as to avoid serious safety accidents that are caused by the failure of OVPS.

## 1. Introduction

Precision, as one of the most important performance indicators of spindle bearings, has a significant impact on the normal operation of machine tools. Super-precision spindle bearings (SPSB) refer to spindle bearings with low vibration, a wide speed range, a high rotational accuracy, low heating, high system rigidity, and low noise. SPSB works at the OVPS, which is the basis for a machine tool to achieve optimal performance. The failure of MTSB are not only due to fatigue. A variety of performance failures, such as stuck, sintering, plastic deformation, crack, or fracture may occur before fatigue failure [1,2,3]. The probability distribution of these failure modes is unknown, and the characteristic data of the spindle bearings are scarce. In particular, the non-linear dynamic contact and collision between the parts inside the spindle bearings, the non-linear damage, viscous-temperature and viscous-pressure characteristics of lubricating medium, and the accuracy loss, all present uncertain and non-linear characteristics [4,5,6,7]. These uncertainties bring new challenges to analyze the degradation trend of vibration performance. Therefore, it is of great significance to evaluate the degradation process of vibration performance dynamically before the MTSB fail.

**Figure 1:**
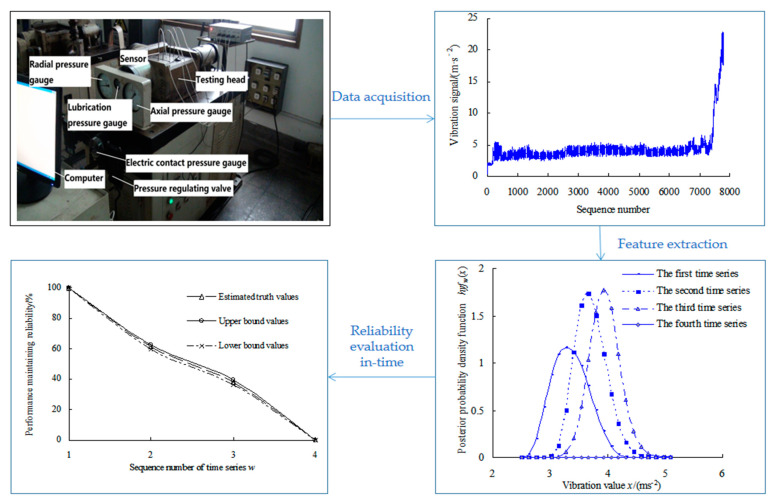
Performance degradation and reliability evaluation process of rolling bearings.

When analyzing the degradation process of bearings, the mathematical statistical methods generally assume that the amount of data are limited, and then extract characteristic parameters of bearings [8,9,10]. Based on the vibration signal collected, Ye et al. [11] used the maximum entropy method to calculate the PDF of bearings, which was regarded as degradation characteristics. Then, the Bayesian method, the bootstrap method and the maximum entropy method were fused to evaluate the reliability of bearings in service. Its performance degradation and reliability evaluation process are shown in Figure 1.

However, due to the differences of experimental data samples caused by random factors and interference factors in the experimental process, or the incompleteness of information of data samples, it is difficult to represent the entire distribution range of bearing life accurately. For two sets of bearings with the same batch and model, the prediction results may also be different, that is, the generalization ability and stability of the model cannot be guaranteed. In order to reduce the impact of random and interfering factors on the analysis results, many experts and scholars at home and abroad preprocess the experimental data. Huang et al. [12] fused empirical mode decomposition method and Hilbert transform method to analyze the time-frequency spectrum of signals, making up for the shortcomings of traditional statistical theory in analyzing nonlinear and non-stationary data. In order to avoid multiple spline curve fitting in the process of empirical mode decomposition, Feldman [13] proposed a time-varying vibration decomposition method based on Hilbert transform. In order to not affect the accuracy of condition monitoring and fault diagnosis, Guo et al. [14] proposed a signal compression method based on integrated empirical mode decomposition, which adaptively decomposes the vibration signal into signal components with different frequency bands. These preprocessing methods may reduce the influence of random and interference factors, while also filtering out some useful information, resulting in “distortion” of the analysis results, which in turn leads to the inability to evaluate the degradation process of bearings effectively.

Traditional machine learning and deep learning, as methods widely used in data preprocessing, have their own advantages and disadvantages. In the field of traditional machine learning, SVM has a low error rate of generalization, a low computational overhead, and it is easy to interpret its results [15,16]. However, SVM is too sensitive to parameters of kernel functions. The idea of KNN is simple, with no assumptions about data input, but its disadvantage is that the computational complexity is high [17]. The logistic regression method has low computational cost, and it is easy to understand and implement [18]. However, it is prone to underfitting and its classification accuracy is not high. The decision tree is insensitive to missing intermediate values, and can handle irrelevant feature data [19]. It is better than KNN in understanding the inherent meaning of the data. Its disadvantage is that it is prone to overfitting and the construction process is time-consuming.

Deep learning and traditional machine learning are similar in data preprocessing. The core difference lies in the feature extraction process, where deep learning does not require manual extraction and the extraction process is performed by a machine [20,21,22,23]. Although deep learning can learn the features of patterns automatically and achieve high recognition accuracy, the prerequisite is that amounts of data are provided. For a limited amount of data, deep learning algorithms cannot estimate the laws of data without bias. The weight parameters of CNN are less than those of DNN connected fully, which makes the training speed of CNN model faster and is not prone to overfitting [24]. Meanwhile, CNN requires less data to train. The disadvantage is that it requires large sample size and parameter adjustment, and its physical meaning is also unclear. RNN cannot solve the problem of long-term dependence, while LSTM implements temporal memory function and prevents gradient disappearance. At the same time, LSTM can better handle the tasks of time series than CNN. In addition, it also solves the long-term dependency problem [25,26,27]. However, the model structure of LSTM is complex relatively, and its training process is more time-consuming than CNN. The characteristics of RNNs determine that they cannot parallelize data well. It is also difficult for LSTM to handle longer data sequences.

Vibration data collected through the experiment vary with time, which can be regarded as a non-linear time series. From the beginning of service to the failure of accuracy, the vibration performance varies continuously, forming multiple time series. The intrinsic series refers to the time series with OPS. According to the time series of MTSB, the degradation process of accuracy is defined as a Poisson process, and the degradation probability is taken as a parameter of the counting process [28]. Influenced by many factors, the degradation process of bearings has the characteristics of non-linear dynamics [29,30]. For each time series of accuracy, the degradation probability relative to the intrinsic series also has the characteristics of non-linearity and diversity. This causes a dynamic change in information. In particular, the degradation probability functions of accuracy vary with time and environmental factors. In view of this, a polynomial is used to fit the parameters, and the data samples of the degradation probability of OVPS are obtained for each time series. Since it is difficult to obtain enough original information of degradation probability in a short time, the grey bootstrap method is used to generate a large set of sample data [31].

Information fusion technology and the real-time update method can improve the prediction accuracy, but most of the existing fusion methods focus on the fusion of homogeneous information collected by the sensors with same type. They rarely consider the fusion of heterogeneous information, especially the fusion and prediction of event single value data and waveform data monitored, which needs further in-depth study [32,33]. In addition, the current real-time update methods basically take Bayesian method as the theoretical framework. They can only apply the observed data to update the priori probability distribution, but cannot utilize other available information such as moments of parameters or functions of moments.

On the basis of this, a dynamic evaluation model is established to study the performance degradation process for MTSB. This model considers the interference of random factors during the service process of MTSB. First, the vibration signal are processed into segments to obtain the time series with OVPS based on rolling average method. The maximum entropy method is used to calculate the PDF of intrinsic sequence. Based on the Poisson counting principle, the variation probabilities of OVPS of each time series are calculated. Considering the interference of random factors, the small data sample of variation probability is obtained for each time series by polynomial fitting method. The grey bootstrap method (namely, GBM (1,1)) is applied to generate enough data samples of variation probability, so as to obtain the estimated true value, estimated interval and dynamic uncertainty of variation probability. The PMR and PMRR of OVPS of MTSB are calculated according to Poisson process theory. Experimental verification is utilized to verify that the proposed model can be used effectively to estimate the failure degree of OVPS to monitor and analyze the performance degradation process of MTSB.

**Figure 2:**
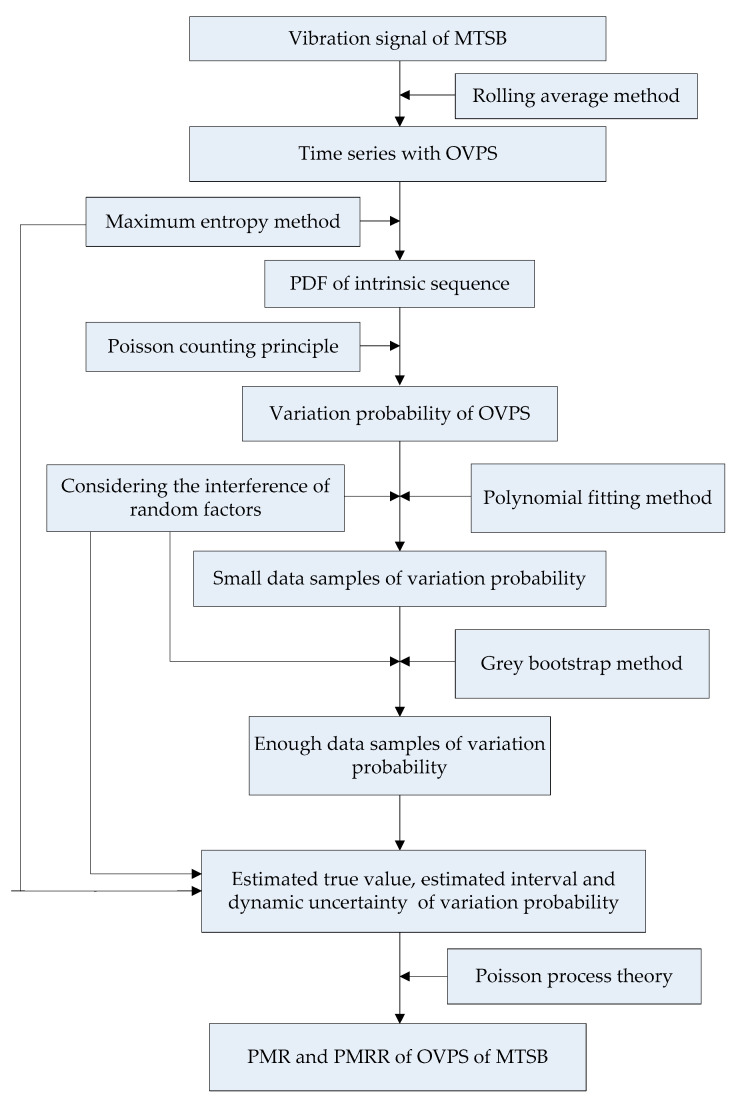
Flow diagram of proposed method.

The flow diagram of proposed method is shown in Figure 2.

## 2. Mathematical Models

Based on the maximum entropy method and the Poisson counting principle, the variation probability of the OVPS is calculated for MTSB. The small data sample of variation probability is obtained by polynomial fitting for each time series. The estimated true value curve and the upper- and lower-bound curves of variation probability are obtained by fusing the grey bootstrap method into the maximum entropy method. The VPMR is calculated based on the Poisson process, which is used to dynamically evaluate the failure degree of OVPS for MTSB.

### 2.1. Calculating Variation Probability of OVPS

During the service period of the spindle bearings, *r* time sequences are obtained by periodic sampling vibration acceleration signals of spindle bearings. The time series collected after initially wearing is considered to be the intrinsic sequence, which is marked as the first time series and expressed by the vector ***X***_1_ as

(1) $$X_{1}=(x(1),x(2),\ldots ,x(k),\ldots ,x(N)).$$

where *x*(*k*) is *k*th performance data in the intrinsic sequence; *k* is the order number of performance data in intrinsic sequence, *k* = 1, 2, 3, …, *N*; *N* is the total number of performance data in the intrinsic sequence.

With the change of time variable, vibration acceleration data are collected continuously, and the *n*th time series vector ***X****_n_* is obtained.

(2) $$X_{n}=(x_{n}(1),x_{n}(2),\ldots ,x_{n}(k),\ldots ,x_{n}(N)).$$

where *x_n_*(*k*) is the *k*th performance data of the *n*th time series; *n* is the order number of time series, *n* = 1, 2, 3, …, *r*.

The maximum entropy method can make the optimal estimation for the unknown probability distribution with minimal subjective bias. Lagrange multipliers are introduced in the process of solving probability distributions, so the problem of solving probability distribution is transformed into the problem of solving Lagrange multipliers. For the convenience of description, the continuous variable *x* is used to express the discrete variable *x*(*k*) in the intrinsic sequence.

According to the maximum entropy method, the probability density function should meet the condition that the value of entropy function *H*(*x*) is the maximum.

(3) $$H(x)=-\int_{S}f(x)\ln f(x)dx$$

where *f*(*x*) is the probability density function of continuous variable *x*; ln*f*(*x*) is the logarithm of the probability density function *f*(*x*); *S* is the feasible domain of the performance random variable *x*, *S* = [*S*_1_, *S*_2_]; *S*_1_ is the lower-bound value of the feasible domain, and *S*_2_ is the upper-bound value of the feasible domain.

Then, the Lagrange multiplier method is used to solve this problem by adjusting the probability density function *f*(*x*) to maximize the value of entropy function *H*(*x*) [11]. Assume *H* is the Lagrange function.

(4) $$\bar{H}=H(x)+(c_{0}+1)[\int_{S}f(x)dx-1]+\sum_{i=1}^{j}c_{i}[\int_{S}x^{i}f(x)dx-m_{i}]$$

where *i* is the order number of origin moment, *i* = 1, 2, …, *j*, usually *j* = 5; *m_i_* is the *i*th order origin moment*, m*_0_ = 1; *x^i^* is the coefficient of the function *f*(*x*); *c_i_* is the (*i* + 1)th Lagrange multiplier and *c*_0_ is the first Lagrange multiplier, *i* = 1, 2, …, *j*.

The probability density function *f*(*x*) of data samples can be expressed as

(5) $$f(x)=\exp(c_{0}+\sum_{i=1}^{j}c_{i}x^{i})$$

With

(6) $$c_{0}=-\ln(\int_{S}\exp(\sum_{i=1}^{j}c_{i}x^{i})dx)$$

The other *j* Lagrange multipliers should satisfy that

(7) $$1-\frac{\int_{S}x^{i}\exp(\sum_{i=1}^{j}c_{i}x^{i})dx}{m_{i}\int_{S}\exp(\sum_{i=1}^{j}c_{i}x^{i})dx}=0$$

To ensure the convergence of solution, the original data interval is mapped to interval [−e, e] by the substitution of variable. The sample data are divided into *ξ* groups in the incremental order, and the histogram can be drawn. At the same time, the values *z_μ_* and frequency *Γ_μ_* can be calculated, and *u* = 2, 3, …, *ξ* + 1. Then, the histogram is extended into (*ξ* + 2) group and let *Γ*_1_ = *Γ_ξ_*_+2_.

Let

(8) ψ=ax+b

where *ψ* is the variable to be transformed, *ψ* ∈ [−e, e]; *a* and *b* are the mapping parameters; e has a value of 2.71828.

(9) $$x=\frac{\psi -b}{a}$$

The mapping parameters *a* and *b* can be calculated based on d*x* = d*ψ*/*a*.

(10) $$a=\frac{2e}{z_{\xi +2}-z_{1}}$$

(11) $$b=e-az_{\xi +2}$$

Therefore, the probability density function *f*(*x*) can be transformed into

(12) $$f(x)=\exp[c_{0}+\sum_{i=1}^{j}c_{i}{\left(ax+b\right)}^{i})]$$

Set a significant level *α*, *α* ∈ [0, 1], so the confidence level *P* is given by

(13) α=(1−P)×100%

The maximum entropy estimated interval is [*X*_L_, *X*_U_] for a given confidence level *P*, and the lower-bound value *X*_L_ should meet that *X*_L_ *= X*_α/2_.

And

(14) $$\frac{1}{2}\alpha =\int_{S_{1}}^{X_{L}}f(x)dx$$

The upper-bound value should meet that *X*_U_ *= X*_(1−α)/2_. And

(15) $$\frac{1}{2}\alpha =\int_{X_{U}}^{S_{2}}f(x)dx$$

So the maximum entropy estimated interval for continuous variable *x* can be given by

(16) $$\left[X_{L},X_{U}\right]=[X_{\frac{\alpha }{2}},X_{1-\frac{\alpha }{2}}]$$

According to Equation (16), the maximum entropy estimated interval [*X*_L1_, *X*_U1_] can be calculated for the intrinsic time series, where *X*_L1_ is the lower-bound value and *X*_U1_ is the upper-bound value of the maximum entropy estimated interval of the intrinsic time series.

Based on the Poisson counting principle, record the number *N_n_* that performance data of the *n*th time series is outside the estimated interval [*X*_L1_, *X*_U1_]. Then, the variation frequency *λ_n_* can be given as Equation (17) for the *n*th time series.

(17) $$\lambda_{n}=\frac{N_{n}}{N}$$

With

(18) $$N_{n}=N_{n1}+N_{n2}.$$

where *N_n_*_1_ is the number showing that performance data are less than *X*_L1_ for the *n*th time series; *N_n_*_2_ is the number showing that performance data are more than *X*_U1_ for the *n*th time series; *n* is the sequence number of time series, *n* = 1, 2, 3, …, *r*.

### 2.2. Calculating Estimated Truth Value and Estimated Interval of Variation Probability

The least-squares method is used to fit the variation probability with different order polynomials. The fitting effect depends on the correlation coefficient R^2^. The closer the correlation coefficient R^2^ is to 1, the better the polynomial fitting effect. If the correlation coefficient R^2^ is less than 0.8, the fitting effect is worse, so the polynomial will not be used in the later analysis process.

The polynomial function *G_q_*(*λ*) is given by

(19) $$G_{q}\left(\lambda \right)=p_{q0}\lambda^{0}+p_{q1}\lambda^{1}+p_{q2}\lambda^{2}\ldots +p_{q\gamma }\lambda^{\gamma }+\ldots +p_{qq}\lambda^{q};\gamma =0,1,2,\ldots ,q;q=1,2,\ldots ,6;$$

where *G_q_*(*λ*) is the *q*th order polynomial; *q* is the order number of polynomial function; *p_qγ_* is the coefficient of the power function *λ^γ^*.

According to the above polynomial function models, small data samples of variation probability of OVPS are obtained for each time series.

(20) $$Y\left(n\right)=\left(y_{n}\left(1\right),y_{n}\left(2\right),\ldots ,y_{n}\left(6\right)\right)=\left(y_{n}\left(u\right)\right);u=1,\;2,\ldots ,6;n=1,2,\ldots ,r;$$

where ***Y***(*n*) is the data sample of variation probability of OVPS for the *n* time series; *y_n_*(*u*) is the *u*th data in the variation probability data sample for the *n* time series.

Using the bootstrap method, *B* bootstrap re-sampling samples of size *z*, namely the bootstrap re-sampling samples ***V***_Bootstrap_, can be obtained by an equiprobable sampling as

(21) $$V_{\mathrm{Bootstrap}}=\left(V_{1},V_{2},\ldots ,V_{\beta },\ldots ,V_{B}\right)$$

where ***V****_β_* is the *β*th bootstrap re-sampling sample, *β* = 1, 2, …, *B*; *B* is the times of the bootstrap re-sampling, and also the number of bootstrap samples, with

(22) $$V_{\beta }=\left[v_{\beta }(\Theta )\right];\Theta =1,2,\ldots ,z$$

where *v_β_*(Θ) is the Θth data in the *β*th bootstrap re-sampling sample.

According to the grey model GM (1,1) [34], suppose the first-order accumulated generating operator (1-AGO) of ***V****_β_* is given by

(23) $$Y_{\beta }=\left[y_{\xi \beta }\left(u\right)\right]=\sum_{j=1}^{\Theta }v_{\xi \beta }\left(j\right)$$

The grey generated model can be described as the differential equation as follows:

(24) $$\frac{dy_{\xi \beta }\left(u\right)}{du}+c_{1}y_{\xi \beta }\left(u\right)=c_{2}$$

where *u* is the time variable, and *c*_1_ and *c*_2_ are the coefficients to be estimated.

Use the increment to replace the differential, viz.,

(25) $$\frac{dy_{\xi \beta }\left(u\right)}{du}=\frac{\Delta y_{\xi \beta }\left(u\right)}{\Delta u}=y_{\xi \beta }\left(u+1\right)-y_{\xi \beta }\left(u\right)=v_{\xi \beta }\left(u+1\right)$$

where Δ*u* is equal to the unit interval, 1. Furthermore, assume the generated vector of the mean series as follows

(26) $$Z_{\beta }=\left[z_{\beta }(u)\right]=\left[0.5y_{\xi \beta }(u)+0.5y_{\xi \beta }(u-1)\right]$$

The least-squares solution with the initial condition *y_ξβ_*(1) = *v_ξβ_*(1) is given by

(27) $${\hat{y}}_{\xi \beta }\left(z+1\right)=\left(v_{\xi \beta }\left(1\right)-c_{2}/c_{1}\right)e^{{-c}_{1}z}+c_{2}/c_{1}$$

where the coefficients *c*_1_ and *c*_2_ are as follows

(28) $${\left(c_{1},c_{2}\right)}^{T}={\left(D^{T}D\right)}^{-1}D^{T}{\left(V_{\beta }\right)}^{T}$$

with

(29) $$D={\left(-Z_{\beta },I\right)}^{T}$$

(30) $$I=\left(1,1,\ldots ,1\right)$$

According to the inverse AGO [34], the *β*th generated data are expressed as

(31) $$\hat{\nu }\left(z+1\right)={\hat{y}}_{\xi \beta }\left(z+1\right)-{\hat{y}}_{\xi \beta }\left(z\right)-c$$

Therefore, *B* generated data for the vibration performance can be obtained as

(32) $$Y_{B}=\left(w_{1},w_{2},\ldots ,w_{\beta },\ldots ,w_{B}\right)=\left({\hat{\nu }}_{1}\left(z+1\right),{\hat{\nu }}_{2}\left(z+1\right),\ldots ,{\hat{\nu }}_{\beta }\left(z+1\right),\ldots ,{\hat{\nu }}_{B}\left(z+1\right)\right)$$

where *w_β_* is the *β*th generated data.

The maximum entropy method is used to calculate the probability density function of the generated sample ***Y****_B_*. According to the probability density function, the true value and upper- and lower-bound values are estimated for the data sample of variation probability of each time series.

The probability density function of variation probability data sample can be calculated as

(33) $$f(\lambda_{n})=\exp[c_{0n}+\sum_{i=1}^{j}c_{in}{\left(a_{n}\lambda_{n}+b_{n}\right)}^{i})]$$

The estimated true value of the variability probability is obtained as

(34) $$\lambda_{n0}=\int_{S}\lambda_{n}f(\lambda_{n})d\lambda_{n}$$

Set a significant level and let *α* ∈ (0, 1). The maximum entropy estimated interval is given as

(35) $$\left[\lambda_{nL},\lambda_{n}{}_{U}]=[\lambda_{n}{}_{\frac{\alpha }{2}},\lambda_{n}{}_{{{}_{1-}}_{\frac{\alpha }{2}}}\right]$$

where *λ_n_*_L_ is the lower-bound value and *λ_n_*_U_ is the upper-bound value of the variation probability data sample for the *n*th time series.

### 2.3. Evaluation of the Uncertainty of Variation Probability

The fluctuation range of variation probability can be expressed as

(36) $$U_{\lambda }{}_{{}_{n}}=\lambda_{n}{}_{U}-\lambda_{n}{}_{L}$$

where *U_λn_* is the estimated uncertainty of variation probability, namely, the instantaneous uncertainty at the confidence level *P*.

The number *η* is calculated for variation probability sample data more than the upper-bound value *λ_n_*_U_ based on the Poisson counting process, and the reliability of evaluation result is defined as

(37) $$P_{R}=\left(1-\eta /r\right)\times 100\%$$

where *P_R_* is the reliability of the polynomials fitting effect using the least-squares method.

Define

(38) $$U_{\mathrm{mean}}=\left(1/r\right)\sum_{n=1}^{r}U_{\lambda_{n}}|{}_{P_{R}=100\%}$$

where *U*_mean_ is the dynamic average uncertainty; |*_PR_*_=100%_ stands for that the calculation process is under the condition of *P_R_* = 100% [9].

### 2.4. Evaluation of PMR and PMRR

Any counting process can be described by the Poisson process [28] as

(39) $$Q=\exp(-\theta \xi )\frac{{\left(\theta \xi \right)}^{e}}{e!}$$

where *ξ* stands for the time variable with *ξ* = 1, 2, 3, …, and *ξ* ≥ 1; *e* is the number of occurring failure events with *e* = 0, 1, 2, 3, …, namely, the serious variation in working condition that may cause the OVPS failure; *Q* is the probability of failure events occurring *e* times. Thus, the reliability *R* for events occurring can be obtained by the Poisson process.

When solving for the PMR of bearings working at the OVPS, let *e* = 0, viz., which indicates that *R* is the probability before the OVPS fails. Let *ξ* = 1, which indicates that *R* is the PMR of time series in current time. According to Equation (40), PMR can be expressed as

(40) $$R\left(\lambda_{n}\right)=\exp\left(-\lambda_{n}\right)$$

where *R*(*λ_n_*) is a function of the variation probability *λ_n_*.

The upper- and lower-bound values of variation probability are applied into the reliability Equation (41), so the estimated true values *R*_0_ and upper- and lower-bound intervals [*R*_L_, *R*_U_] are gained for performance maintaining reliability during the time intervals corresponding to the performance time series. The range of variation probability *λ_n_* is [0, 1]. 0 represents the OVPS of bearings without any variation, which is an ideal state and the reliability is 100% for bearing working at the OVPS. 1 represents that the OVPS of rolling bearings fail completely and the performance state is very unreliable. Therefore, if the value of *λ_n_*_L_ is less than 0, let *λ_n_*_L_ = 0 artificially in the process of solving the performance maintaining reliability.

*R*_0_ = exp(−*λ_n_*_0_), *R*_L_ = exp(−*λ_n_*_U_), *R*_U_ = exp(−*λ_n_*_L_) (41)

According to the concept of relative error in measurement theory, performance maintaining relative reliability (PMRR) *d*(*λ_n_*) of MTSB is obtained to characterize the failure degree of OVPS [11].

(42) $$d\left(\lambda_{n}\right)=\frac{R\left(\lambda_{n}\right)-R\left(\lambda_{1}\right)}{R\left(\lambda_{1}\right)}\times 100\%$$

where *R*(*λ*_1_) is the PMR for the intrinsic series of MTSB, *R*(*λ_n_*) is the PMR for the *n*th time series of MTSB; *d*(*λ_n_*) is the PMRR for the *n*th time series of MTSB.

The basic classification principle of degree failure of OVPS for MTSB is as follows:

(1) If *d*(*λ_n_*) is not less than 0%, which shows that the PMR during this period is not less than PMR of OVPS, and it cannot deny that the performance has reached its optimal state; otherwise, it can deny that the performance has achieved its optimal state.
(2) When *d*(*λ_n_*) is less than 0%, if the absolute value of *d*(*λ_n_*) is in (0%, 15%], this indicates that the error between the evaluation value and the optimum value is very small. If the absolute value of *d*(*λ_n_*) is in (15%, 30%], this indicates that the error between the evaluation value and the optimum value is gradually increasing. If the absolute value of *d*(*λ_n_*) is greater than 30%, this indicates that the error between the evaluation value and the optimum value is very large.

Based on that, the degree failure of OVPS for MTSB is divided into S1, S2, S3, S4 for a total of four levels:

S1: If *d*(*λ_n_*) ≥ 0%, it indicates the performance states of MTSB reaches the optimum and has almost no failure possibility.

S2: If *d*(*λ_n_*) ∈ [−15%, 0%), it indicates the performance states of MTSB is normal, and the degree failure of OVPS is very small.

S3: If *d*(*λ_n_*) ∈ [−30%, −15%), it indicates the performance states of MTSB is gradually becoming worse, and the degree failure of OVPS is gradually increasing.

S4: If *d*(*λ_n_*) < −30%, it indicates the performance states of MTSB is worse, and the degree failure of OVPS is very large.

The negative value of *d*(*λ_n_*) indicates the performance states has attenuation, namely, PMR currently is less than PMR during the optimum period, and the positive value indicates no attenuation. The smaller *d*(*λ_n_*) is, the worse the performance states of MTSB is, and the larger the degree failure of OVPS is. Therefore, the time corresponding to *d*(*λ_n_*) = −30%, is the critical time where OVPS becomes poor. Taking corresponding measures before the critical time can avoid serious safety accidents that are caused by the failure of OVPS.

## 3. Experimental Verification

### 3.1. Case 1

**Figure 3:**
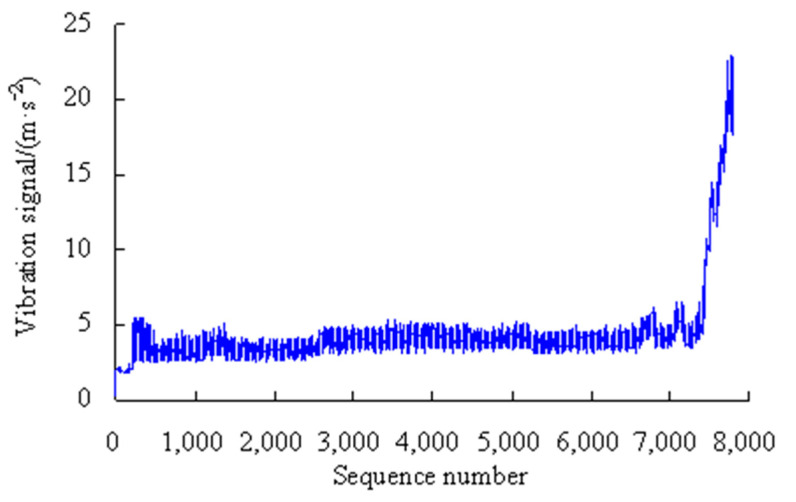
Vibration signals of MTSB.

This is a strength lifetime test on the machine tool spindle bearings, which is conducted at a radial load of 4.58 kN, an axial load of 4.17 kN, and a motor speed 6000 r/min. The vibration acceleration sensor has a measuring range of ±2000 g and a resolution of 0.0001 g. The data-acquisition rate is 20 KHz. The test machine, the bearing used and the vibration data in this case are exactly the same as those of Case 1 in Reference [11]. The vibration signals are automatically collected by the computer control system, as shown in Figure 3.

As shown in Figure 3, based on the rolling average method, the performance degradation stages are divided as shown in Table 1.

From the OVPS of the bearing, there are 7321 vibration signals. Among them, the 473rd to 7472nd data points are divided into 10 segments at 700 data intervals. The 7473rd to 7793rd data are separated into one segment, that is, the 11th segment.

It is worth noting that if the vibration data are divided into fewer segments, the specific variation process of characteristic parameter cannot be obtained. If the vibration data are divided into a large number of segments, it will cause large computational workload and complex calculation process. At the same time, each segment of data contain less information, which makes the calculation error of the probability density function larger. Therefore, the vibration data are divided into ten segments usually in the analysis process.

#### 3.1.1. Variation Probability of OVPS of MTSB

**Figure 4:**
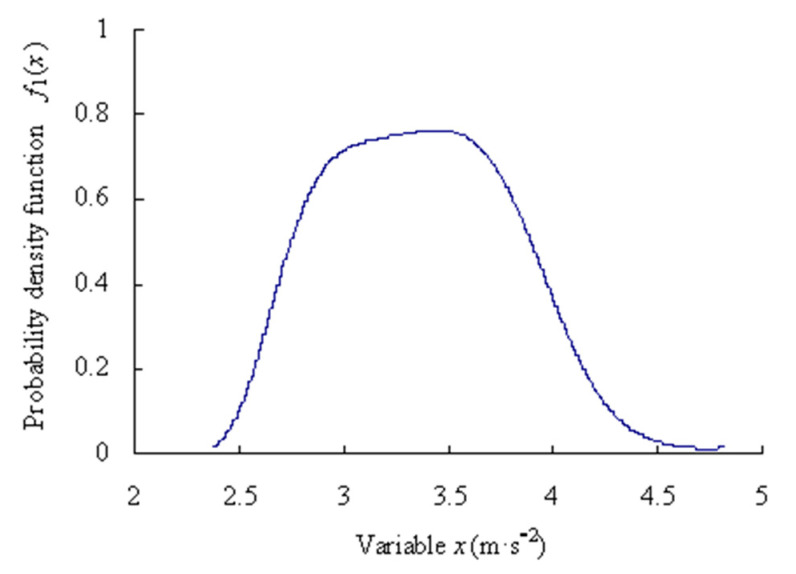
Probability density function of data samples of intrinsic series.

For the first time series, based on the Equations (1)–(12), the origin moments are obtained as [*m*_11_, *m*_21_, *m*_31_, *m*_41_, *m*_51_] = [−0.5266, 1.2312, −1.4627, 3.5077, −5.2016]. Lagrange multipliers are calculated as [*c*_01_, *c*_11_, *c*_21_, *c*_31_, *c*_41_, *c*_51_] = [−0.2975, −0.1981, −0.4370, −0.2928, −0.0111, 0.0430]. Mapping parameter are gained as *a*_1_ = 2.2241; *b*_1_ = −8.0066. The estimated truth function *f*_1_(*x*) is calculated as shown in Figure 4.

Assume that the significance level *α* is 0.01 and the confidence level is *P* = 99%, based on the Equations (13)–(16), the maximum entropy estimated interval of the intrinsic series is [2.4867, 4.5127] m·s^−2^.

**Figure 5:**
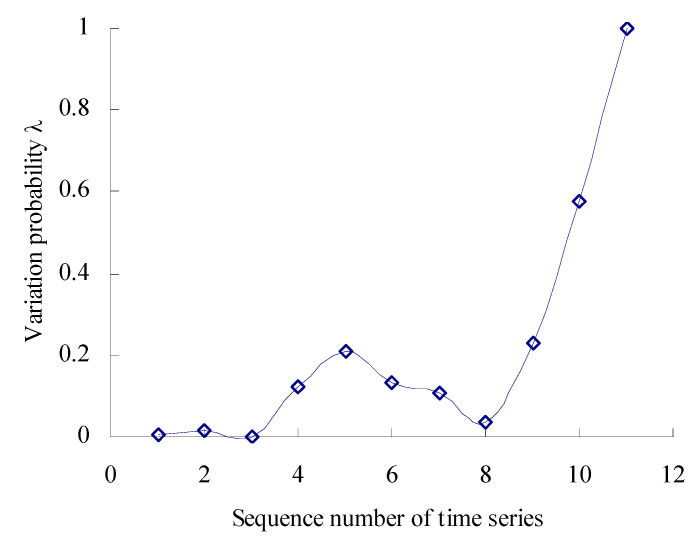
Variation probability curve.

Based on the Equations (17) and (18), the numbers are calculated, respectively, that the 11 data samples fall outside the maximum entropy estimated interval [*X*_L1_, *X*_U1_] of intrinsic time series. According to the Poisson counting principle, the variation frequencies [*λ*_1_, *λ*_2_, *λ*_3_, *λ*_4_, *λ*_5_, *λ*_6_, *λ*_7_, *λ*_8_, *λ*_9_, *λ*_10_, *λ*_11_] are as shown in Figure 5.

As shown in Figure 5, the performance variation probability is non-linear and uncertain–relative to the intrinsic time series. Before the corresponding time interval of the third time series, the variation probability of the MTSB vibration performance is almost zero. Between the corresponding time interval of the third time series and the fifth time series, the variation probability has an increased trend. Between the corresponding time interval of the fifth time series and the eighth time series, the variation probability has a decreasing trend. Between the corresponding time interval of the eighth time series and the eleventh time series, the variation probability has a rapidly increasing trend.

#### 3.1.2. Estimated Truth Value and Estimated Interval of Variation Probability of MTSB

**Figure 6:**
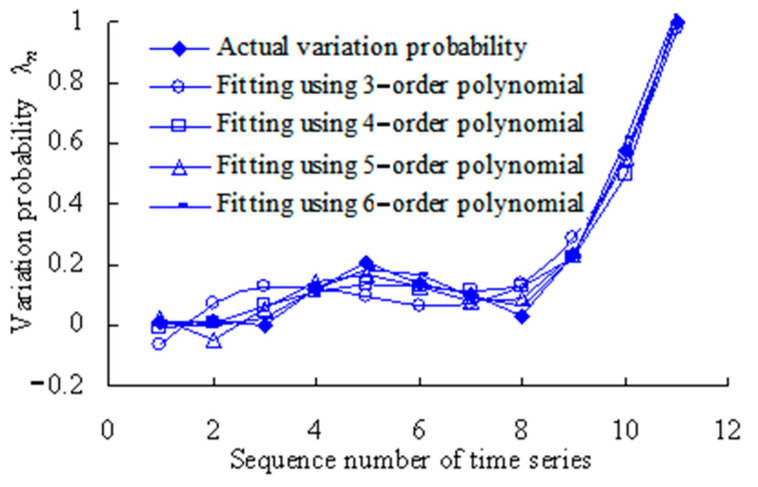
Variation probability curve fitting using least-square method.

The least-squares method is used to fit the variation probability with second-, third-, fourth-, fifth- and sixth-order polynomials. Based on the Equation (19), the fitting results are shown in Table 2 and Table 3, and Figure 6.

The closer the correlation coefficient R^2^ is to 1, the better the polynomial fitting. As shown in Table 4, the correlation coefficient R^2^ for the second-order polynomial is less than 0.8. This shows that the fitting is worse than other higher-order polynomials. Therefore, the second-order polynomial is not used in further analysis.

Figure 6 shows that the fitted variation probability is almost identical with the actual variation probability for the fourth, tenth and eleventh time series. The fitting was performed using the least-square method by the third-order polynomial. With fittings using the least-square method by the fourth-order polynomial, the fitted variation probability values are basically identical with the actual values for the second, fourth, sixth, seventh and ninth time series. The fitted variation probability values are almost identical with the actual variation probability values for the third, sixth, ninth and eleventh time series when fitting was performed using the least-square method by the fifth-order polynomial. When fitting was performed using the least-square method by the sixth-order polynomial, the fitted variation probability values are basically identical with the actual values for the first, third, fourth, fifth, seventh, eighth and ninth time series. In short, the above four polynomials have their own advantages in fitting. Therefore, relevant information should be fully fused to effectively monitor the evolution process of rolling bearing vibration performance.

#### 3.1.3. The Uncertainty of Variation Probability of MTSB

**Figure 7:**
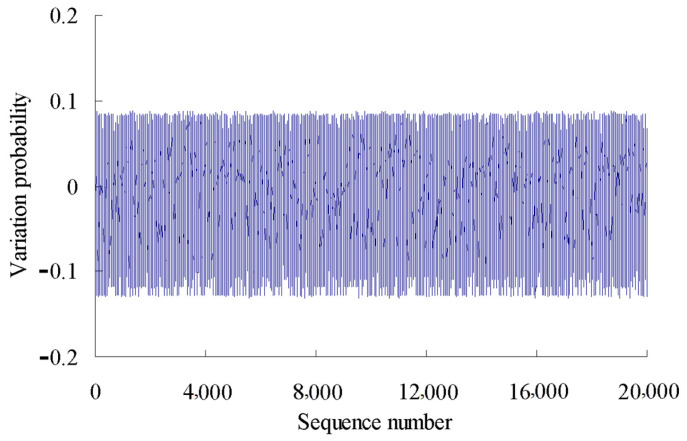
Sample data generated using grey bootstrap method.

The variation probability of the first time series is taken as an example here. According to the grey bootstrap method, the number of bootstrap re-sampling is taken to be *B* = 20,000, and confidence level is taken to be *P* = 95%. Based on the Equations (20)–(32), the grey bootstrap sample ***Y***_Bootstrap_ is obtained by sampling the sample data of fitting the variation probability values using the above four polynomials, as shown in Figure 7.

Based on the maximum entropy method, the origin moments are obtained as [*m*_11_, *m*_21_, *m*_31_, *m*_41_, *m*_51_] = [0.3813, 1.7553, 0.7854, 5.5473, 2.5368] for the grey bootstrap sample of variation probability. Lagrange multipliers are calculated as [*c*_01_, *c*_11_, *c*_21_, *c*_31_, *c*_41_, *c*_51_] = [1.3756, 0.8973, 0.3771, −0.3693, −0.0993, 0.0395] for the grey bootstrap sample of variation probability. Mapping parameter are gained as *a*_1_ = 23.3049; *b*_1_ = 0.5263.

**Figure 8:**
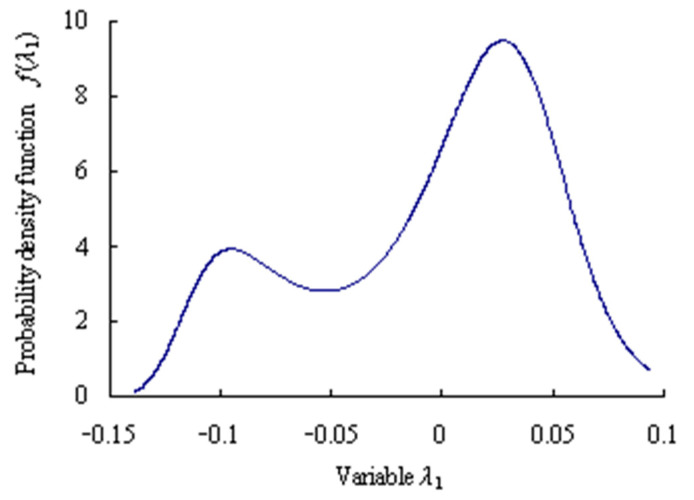
Maximum entropy probability density function of grey bootstrap samples.

The probability density function of the grey bootstrap sample ***Y***_Bootstrap_ is shown in Figure 8.

Assume that the confidence level *α* = 0.05 and the confidence level *P* = 95%. Based on the Equations (33)–(35), the estimated truth value and the estimated interval are obtained as *λ*_10_ = 0.0057 and [*λ*_1L_, *λ*_1U_] = [−0.1299, 0.0711] for the grey bootstrap sample of variation probability of the first time series.

**Figure 9:**
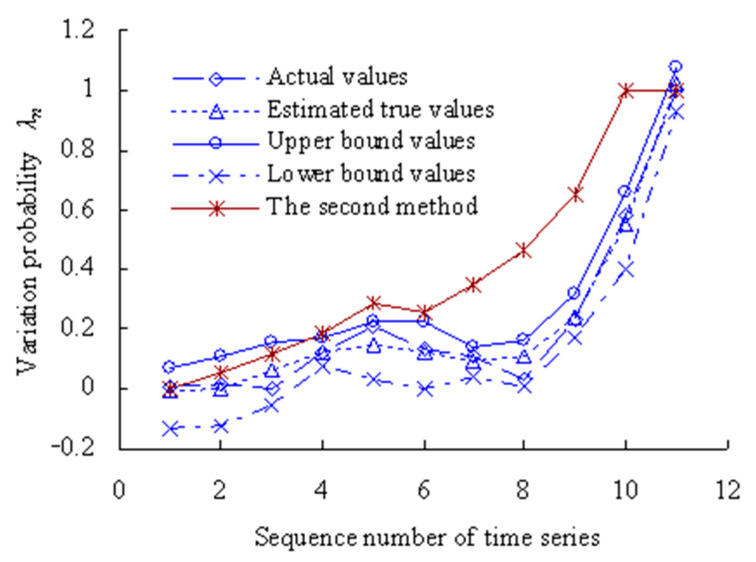
Estimated results of degradation probability of OVPS of MTSB.

The grey bootstrap maximum entropy method is used to obtain the weighted average values, upper- and lower-bound values for the 11 time series under the condition that the confidence level is 0.05, as shown in Figure 9.

Figure 9 shows that the values of variation probability calculated using the method in the Reference [34] (The second method) are larger than those calculated using this method. The variation probability has reached 1 for the 10th time series using the second method, which is less in line with the actual degradation process of OVPS of MTSB. Because the interference of random factors during the service process of MTSB was not taken into account.

Based on the Poisson counting process, the total number is calculated as *η* = 0 for variation probability sample data falling outside the upper-bound value *λ_n_*_U_. That is, the reliability of the evaluation results is *P_R_* = 100%, which satisfies *P_R_* > *P*.

The estimated uncertainties are calculated for the variation probability of rolling bearing vibration performance, as shown in Table 4.

Based on the Equations (36)–(38), the dynamic average *U*_mean_ is calculated for the variation probability of time series as *U*_mean_ = 1.7515/11 = 0.1775.

#### 3.1.4. PMR and PMRR of MTSB

**Figure 10:**
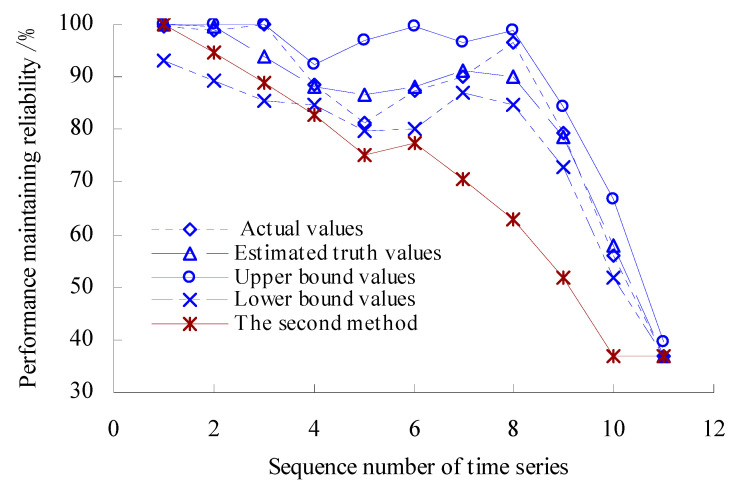
Dynamic evaluation results of PMR.

According to the calculated results of variation probability, the estimated true values *R*_0_ and upper- and lower-bound intervals [*R*_L_, *R*_U_] are found as *R*_0_ = exp(−*λ_n_*_0_), *R*_L_ = exp(−*λ_n_*_U_), *R*_U_ = exp(−*λ_n_*_L_). The range of variation probability *λ* is [0, 1]. Here, 0 represents the OVPS of bearings without any variation, which is an ideal state with the reliability of bearing performance at 100%. On the other hand, 1 represents that the OVPS of spindle bearings fail completely and the performance is very unreliable. Therefore, if the value of *λ*_L_ is less than 0, let *λ*_L_ = 0 (artificially) in the process of solving the performance maintaining reliability. Based on the Equations (39)–(41), the dynamic evaluation results of the VPMR are shown in Figure 10.

As shown in Figure 10, the values of PMR remain unchanged before the time point corresponding to the third time series. During the period corresponding to the third time series to the fifth time series, the values of PMR have a decreasing trend. The value of PMR reaches the minimum, that is, 81.17442% for the fifth time series. The values of PMR have an increasing trend during the period corresponding to the fifth time series to the eighth time series. The values of PMR have a rapidly decreasing trend during the period corresponding to the eighth time series to the eleventh time series. Figure 10 shows that the values of PMR calculated using the second method are smaller than those calculated using this method.

The estimated true value curve coincides with the actual value curve for the PMR of spindle bearings, but the estimated true value curve is smoother. Moreover, the upper- and lower-bound curves envelope the actual value curves fully for the PMR, which verifies the rationality of the evaluation model again.

**Figure 11:**
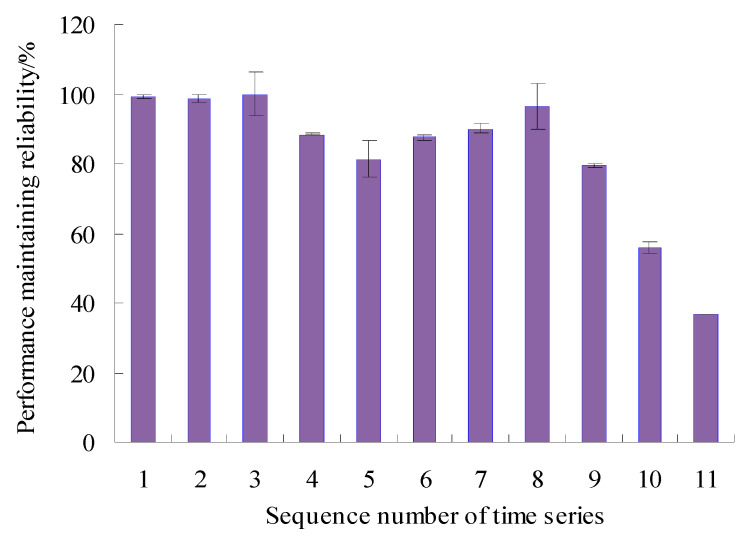
Error bars of PMR.

In order to visualize the difference between the estimated true value and the actual value of the PMR, the error bars of PMR are calculated, as shown in Figure 11.

As can be seen from Figure 11, the maximum error appears in the eighth time series, but is only 6.55%, and the errors were lower than 1.00% for the first, fourth, sixth, ninth, and tenth time series, which shows that the analysis results of the proposed method have good consistency. By analyzing the vibration data in Figure 3, it can be seen that the vibration performance has a sudden change or a large fluctuation during the period corresponding to these time series. Therefore, it is difficult to accurately estimate the values of PMR during the corresponding period.

**Figure 12:**
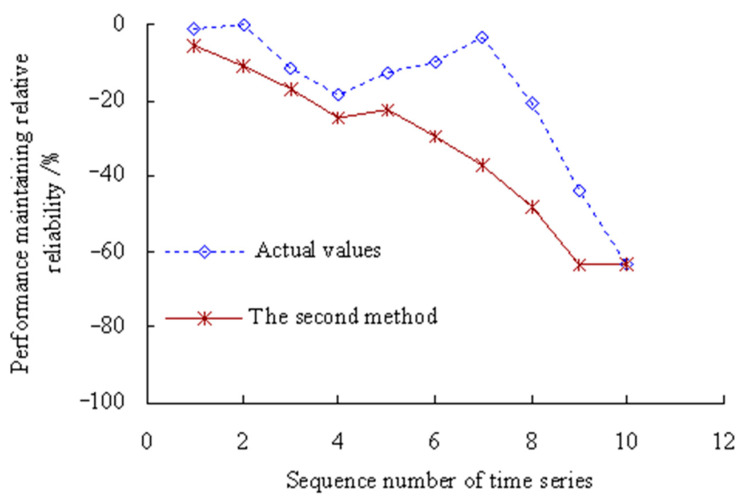
Dynamic evaluation results of PMRR.

According to Equation (42), the dynamic evaluation results of the PMRR are shown in Figure 12.

Figure 12 shows that the actual value of PMRR, *d*(*λ*_1_), *d*(*λ*_3_) ≥ 0%, which shows that the performance states of MTSB reaches the optimum and has almost no failure possibility at the corresponding stage; *d*(*λ*_2_), *d*(*λ*_7_), *d*(*λ*_8_) ∈ [−15%, 0%), which shows that the performance states of MTSB is normal, and the degree failure of OVPS is very small; *d*(*λ*_4_), *d*(*λ*_5_), *d*(*λ*_6_), *d*(*λ*_9_) ∈ [−30%, −15%), which shows that the performance states of MTSB is gradually becoming worse, and the degree failure of OVPS is gradually increasing; *d*(*λ*_10_), *d*(*λ*_11_) < −30%, which shows that the performance states of MTSB is worse, and the degree failure of OVPS is very large. Thus, taking appropriate remedial measures are necessary steps before 6773 min, which can avoid serious safety accidents that are caused by the failure of OVPS.

Figure 12 also shows that the values of PMRR calculated using the second method are smaller than those calculated using this method. The values of PMRR, *d*(*λ*_7_), *d*(*λ*_8_), *d*(*λ*_9_), *d*(*λ*_10_), *d*(*λ*_11_) < −30%, which shows that the degree failure of OVPS is very large after the corresponding time period of the 7th time series. However, this is less in line with the actual degradation process of OVPS of MTSB. Because the interference of random factors during the service process of MTSB was not taken into account.

### 3.2. Case 2

**Figure 13:**
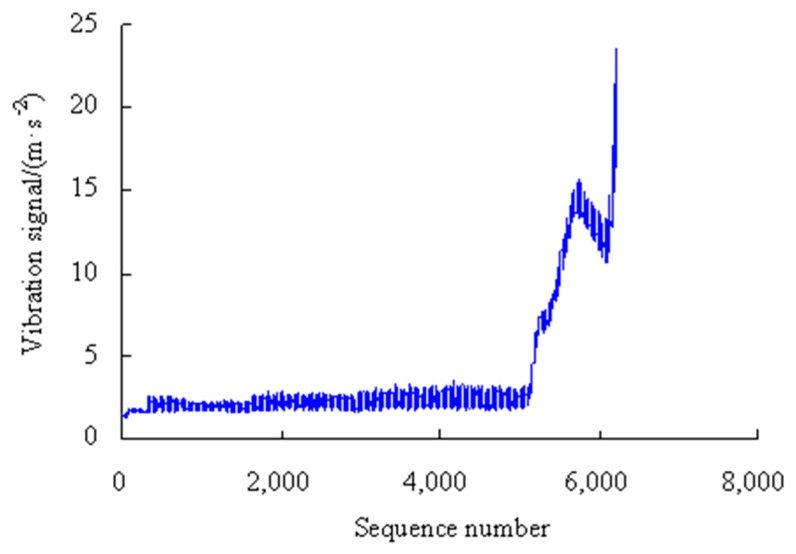
Vibration signals of bearing performance (Case 2).

The test machine and the bearing used in this case are exactly the same as those of Case 1. The vibration data are shown in Figure 13 by changing the test conditions of the motor to a speed of 4000 r/min, an axial load of 4.17 kN, and a radial load of 4.58 kN.

From the OVPS of the bearing, there are 5883 vibration signals. Among them, the 334th to 5933rd data points are divided into seven segments at 800 data intervals. The 5934th to 6216th data are separated into one segment, that is, the 8th segment.

#### 3.2.1. Variation Probability of OVPS of MTSB (Case 2)

For the first time series, based on the Equations (1)–(12), the origin moments are obtained as [*m*_11_, *m*_21_, *m*_31_, *m*_41_, *m*_51_] = [0.0768, 0.9388, 0.1046, 2.2771, 0.3843]. Lagrange multipliers are calculated as [*c*_01_, *c*_11_, *c*_21_, *c*_31_, *c*_41_, *c*_51_] = [0.5329, 0.3193, −0.3891, −0.1865, −0.0271, 0.0252]. Mapping parameter are gained as *a*_1_ = 4.4481 and *b*_1_ = −9.1186.

Assume that the significance level *α* is 0.05 and the confidence level is *P* = 95%, based on the Equations (13)–(16), the maximum entropy estimated interval of the intrinsic series is [1.5435, 2.5871] m·s^−2^.

**Figure 14:**
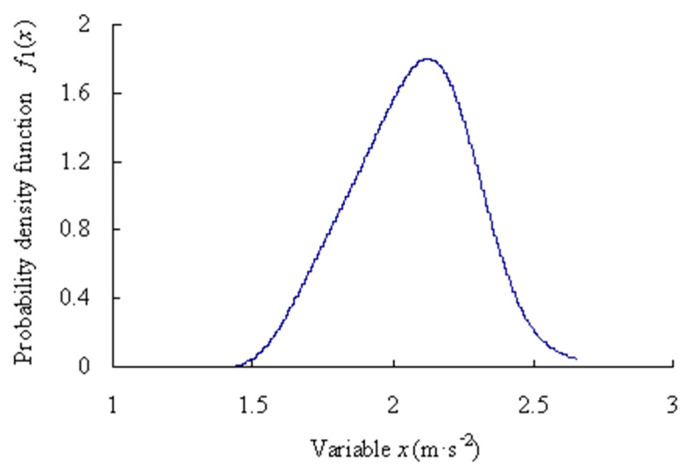
Probability density function of data samples of intrinsic series (Case 2).

The probability density estimated truth function *f*_1_(*x*) is calculated as shown in Figure 14.

**Figure 15:**
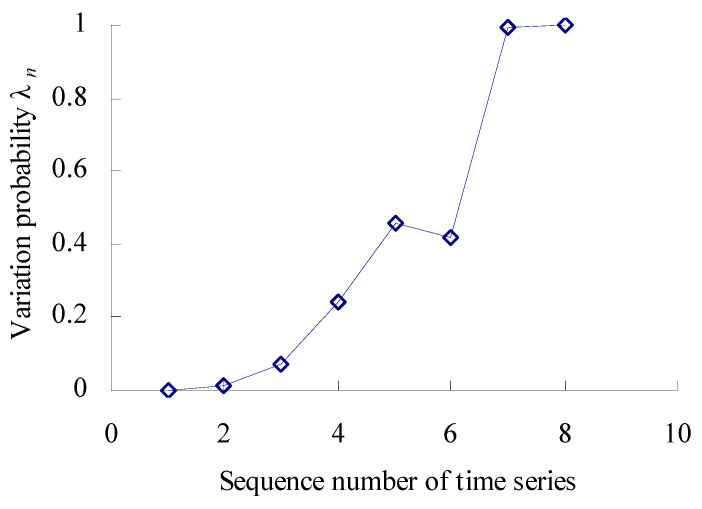
Variation probability curve (Case 2).

Based on the Equations (17) and (18), the numbers are calculated, respectively, that the 8 data samples fall outside the maximum entropy estimated interval of intrinsic time series. According to the Poisson counting principle, the variation frequencies [*λ*_1_, *λ*_2_, *λ*_3_, *λ*_4_, *λ*_5_, *λ*_6_, *λ*_7_, *λ*_8_, *λ*_9_, *λ*_10_, *λ*_11_] = [0, 0.0136, 0.0738, 0.2450, 0.4563, 0.4370, 0.4213, 0.9963, 1], as shown in Figure 15.

As shown in Figure 15, the performance variation probability is non-linear and uncertain–relative to the intrinsic time series. Before the corresponding time interval of the second time series, the variation probability of the bearings’ vibration performance is almost zero. Between the corresponding time interval of the second time series and the fifth time series, the variation probability has an increased trend. Between the corresponding time interval of the fifth time series and the sixth time series, the variation probability has a decreasing trend. Between the corresponding time interval of the sixth time series and the seventh time series, the variation probability has a rapidly increasing trend.

#### 3.2.2. Estimated Truth Value and Estimated Interval of Variation Probability of MTSB (Case 2)

**Figure 16:**
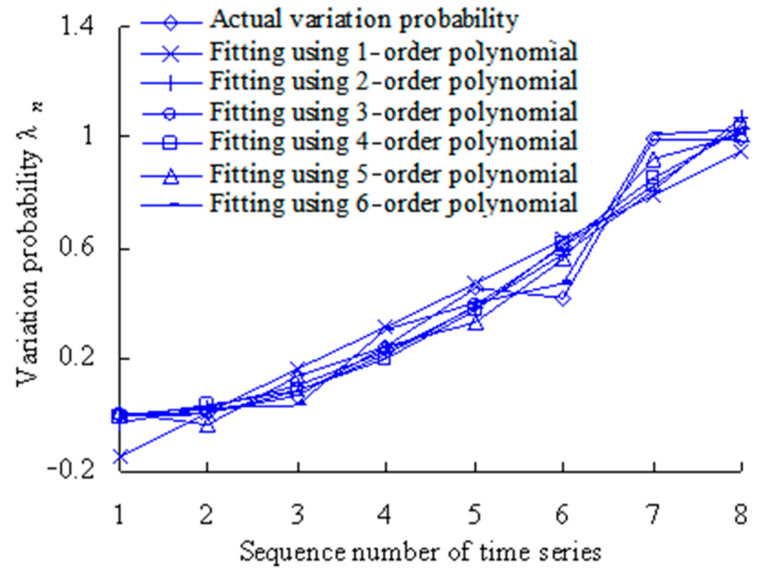
Variation probability curve fitting using least-square method (Case 2).

The least-squares method is used to fit the variation probability with first-, second-, third-, fourth-, fifth- and sixth-order polynomials. Based on the Equation (19), the fitting results are shown in Table 5 and Table 6, and Figure 16.

The closer the correlation coefficient R^2^ is to 1, the better the polynomial fitting. As shown in Table 5, the correlation coefficients R^2^ for all polynomials are more than 0.8. Therefore, the first-order, second-order, third-order, fourth-order, fifth-order, and sixth-order polynomials will be used in further analysis.

Figure 16 shows that the fitted variation probability is almost identical with the actual variation probability for the second and fifth time series. The fitting was performed using the least-square method by the first-order polynomial. With fittings using the least-square method by the second-order polynomial, the fitted variation probability value is basically identical with the actual value for the fourth time series. The fitted variation probability values are almost identical with the actual variation probability values for the first, second and third time series when fitting was performed using the least-square method by the third-order polynomial. When fitting was performed using the least-square method by the fourth-order polynomial, the fitted variation probability value is basically identical with the actual value for the third time series. With fittings using the least-square method by the fifth-order polynomial, the fitted variation probability values are basically identical with the actual values for the fourth, seventh and eighth time series. The fitted variation probability values are almost identical with the actual variation probability values for the first, fifth, sixth, seventh and eighth time series when fitting was performed using the least-square method by the sixth-order polynomial. In short, the above six polynomials have their own advantages in fitting. Therefore, relevant information should be fully fused to effectively monitor the evolution process of rolling bearing vibration performance.

#### 3.2.3. Uncertainty of Variation Probability of MTSB (Case 2)

**Figure 17:**
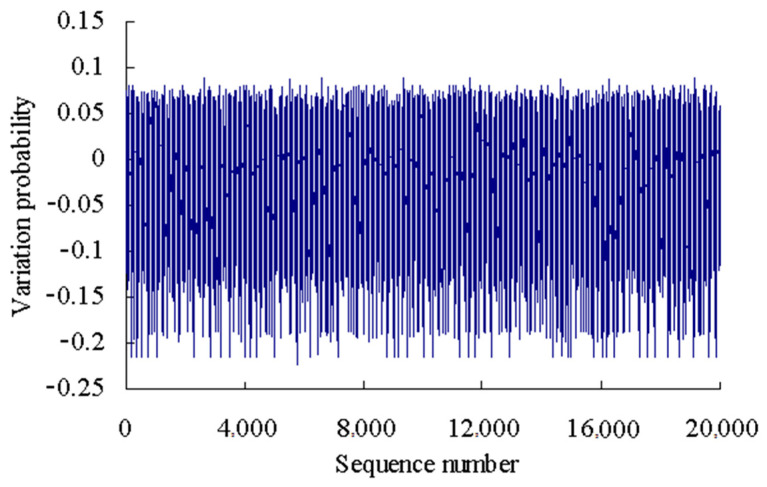
Sample data using grey bootstrap method (Case 2).

The variation probability of the first time series is taken as an example here. According to the grey bootstrap method, the number of bootstrap re-sampling is taken to be *B* = 20,000, and confidence level is taken to be *P* = 95%. Based on the Equations (20)–(32), the grey bootstrap sample ***Y***_Bootstrap_ is obtained by sampling the sample data of fitting the variation probability values using the above six polynomials, as shown in Figure 17.

Based on the maximum entropy method, the origin moments are obtained as [*m*_11_, *m*_21_, *m*_31_, *m*_41_, *m*_51_] = [0.7513, 1.1631, 1.2737, 2.3929, 3.1825] for the grey bootstrap sample of variation probability. Lagrange multipliers are calculated as [*c*_01_, *c*_11_, *c*_21_, *c*_31_, *c*_41_, *c*_51_] = [0.9174, 1.8886, 0.6202, −0.8485, −0.2844, 0.1335] for the grey bootstrap sample of variation probability. Mapping parameter are gained as *a*_1_ = 15.7153; *b*_1_ = 1.0616.

**Figure 18:**
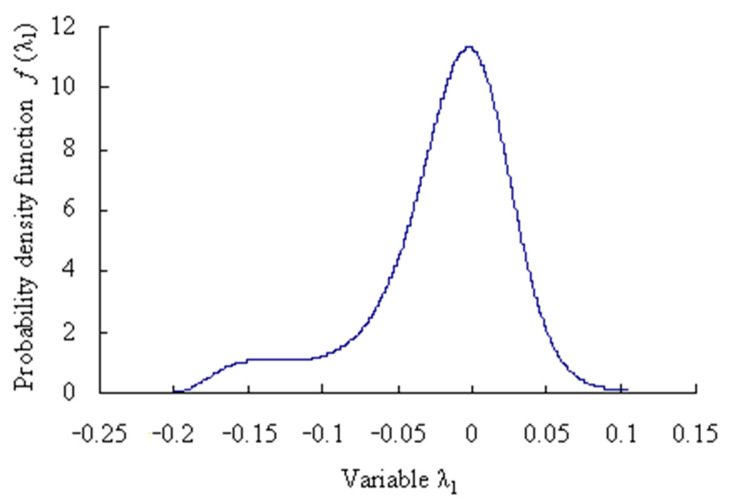
Maximum entropy probability density function of grey bootstrap samples (Case 2).

The probability density function of the grey bootstrap sample ***Y***_Bootstrap_ is shown in Figure 18.

Assume that the confidence level *α* = 0.05 and the confidence level *P* = 95%. Based on the Equations (33)–(35), the estimated truth value and the estimated interval are obtained as *λ*_10_ = −0.0197 and [*λ*_1L_, *λ*_1U_] = [−0.1534, 0.0536] for the grey bootstrap sample of variation probability of the first time series.

**Figure 19:**
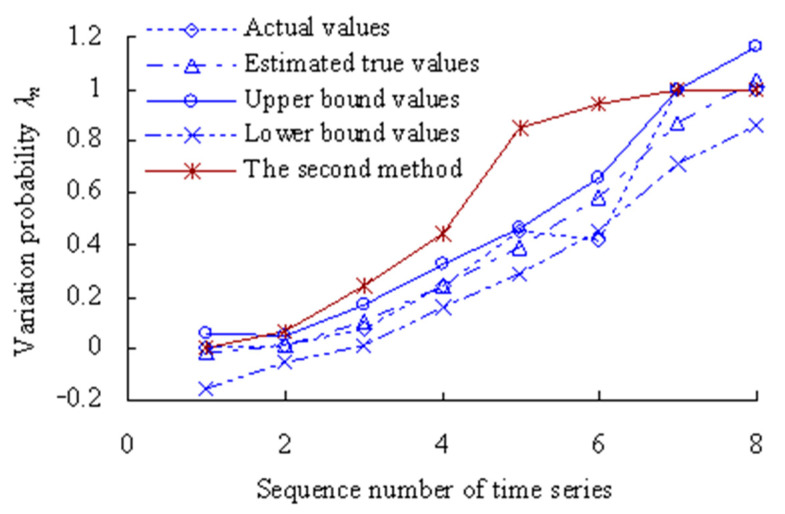
Estimated results of degradation probability of OVPS of MTSB (Case 2).

The grey bootstrap maximum entropy method is used to obtain the weighted average values, upper- and lower-bound values for the 8 time series under the condition that the confidence level is 0.05, as shown in Figure 19.

Figure 19 shows that the values of variation probability calculated using the method in the Reference [34] (The second method) are larger than those calculated using this method. The variation probability has reached 1 for the 7th time series using the second method, which is less in line with the actual degradation process of OVPS of MTSB. Because the interference of random factors during the service process of MTSB was not taken into account.

Based on the Poisson counting process, the total number is calculated as *η* = 0 for variation probability sample data falling outside the estimated upper-bound curve *λ_n_*_U_. That is, the reliability of the evaluation results is *P_R_* = 100%, which satisfies *P_R_* > *P*.

The estimated uncertainty is calculated for the variation probability of rolling bearing vibration performance, as shown in Table 7.

Based on the Equations (36)–(38), the dynamic average *U*_mean_ is calculated for the variation probability of time series as *U*_mean_ = 1.4030/8 = 0.1169.

#### 3.2.4. PMR and PMRR of MTSB (Case 2)

**Figure 20:**
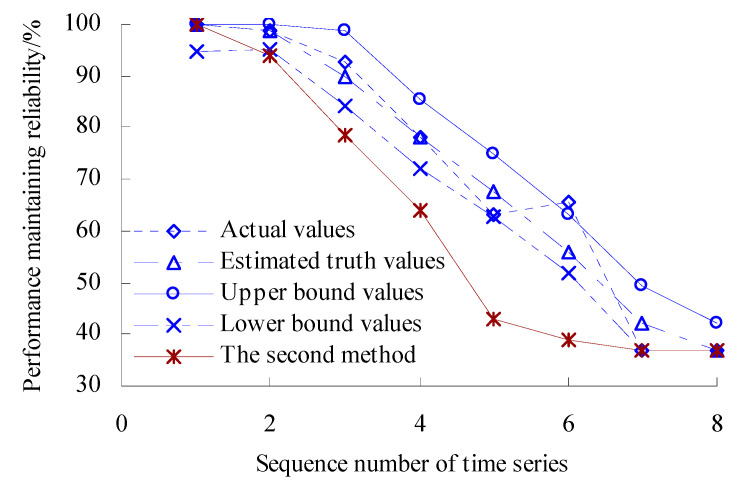
Dynamic evaluation results of PMR (Case 2).

According to the calculated results of variation probability, the estimated true values *R*_0_ and upper- and lower-bound intervals [*R*_L_, *R*_U_] are found as *R*_0_ = exp(−*λ_n_*_0_), *R*_L_ = exp(−*λ_n_*_U_), *R*_U_ = exp(−*λ_n_*_L_). The range of variation probability *λ* is [0, 1]. Here, 0 represents the OVPS of bearings without any variation, which is an ideal state with the reliability of bearing performance at 100%. On the other hand, 1 represents that the OVPS of spindle bearings fail completely and the performance is very unreliable. Therefore, if the value of *λ*_L_ is less than 0, let *λ*_L_ = 0 (artificially) in the process of solving the performance maintaining reliability. Based on the Equations (39)–(41), the dynamic evaluation results of the VPMR are shown in Figure 20.

As shown in Figure 20, the values of VPMR remain unchanged before the time point corresponding to the second time series. During the period corresponding to the second time series to the fifth time series, the values of VPMR have a decreasing trend. The value of VPMR reaches the minimum, that is, 63.36544% for the fifth time series. The values of VPMR have an increasing trend during the period corresponding to the fifth time series to the sixth time series. The values of VPMR have a rapidly decreasing trend during the period corresponding to the sixth time series to the seventh time series. Figure 20 shows that the values of PMR calculated using the second method are smaller than those calculated using this method.

The estimated true value curve coincides with the actual value curve for the VPMR of spindle bearings, but the estimated true value curve is smoother. Moreover, the upper- and lower-bound curves envelope the actual value curves fully for the VPMR, which verifies the rationality of the evaluation model again.

**Figure 21:**
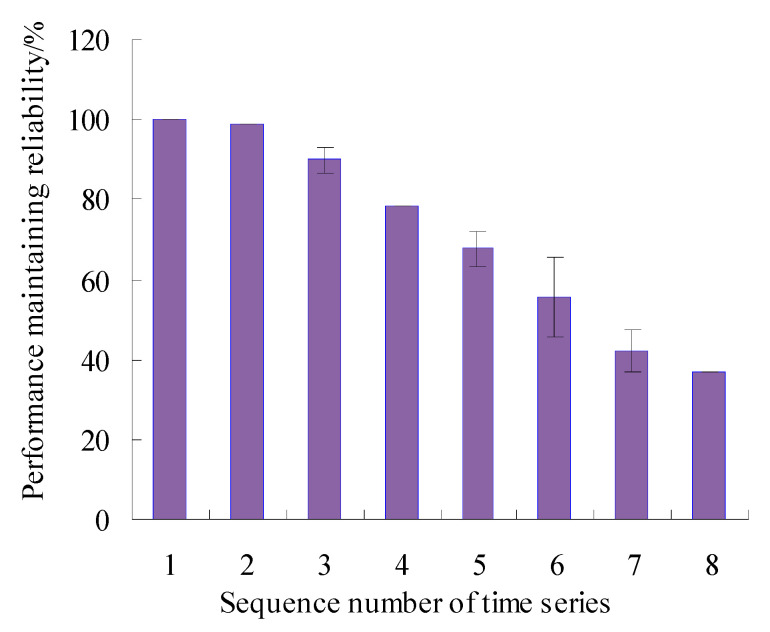
Error bars of PMR (Case 2).

In order to visualize the difference between the estimated true value and the actual value of the VPMR, the error bars of PMR are calculated, as shown in Figure 21.

As can be seen from Figure 21, the maximum error appears in the sixth time series, but is only 9.91%, and the errors were lower than 0.05% for the first, second, fourth and eighth time series, which shows that the analysis results of the proposed method have good consistency. By analyzing the vibration data in Figure 13, it can be seen that the vibration performance has a sudden change or a large fluctuation during the period corresponding to these time series. Therefore, it is difficult to accurately estimate the values of VPMR during the corresponding period.

**Figure 22:**
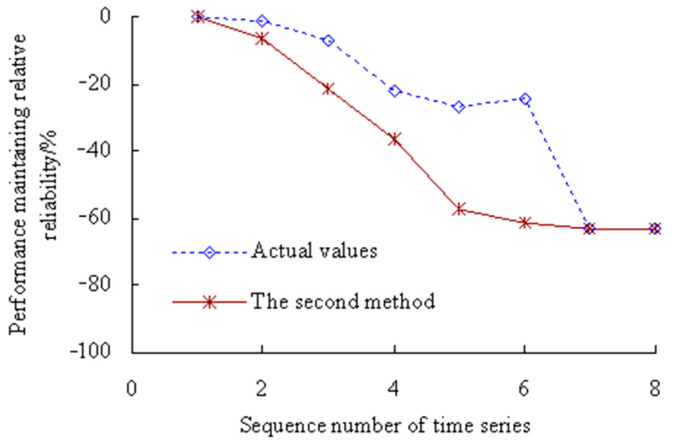
Dynamic evaluation results of PMRR (Case 2).

According to Equation (42), the dynamic evaluation results of the PMRR are shown in Figure 22.

Figure 22 shows that the actual value of PMRR, *d*(*λ*_1_) ≥ 0%, which shows that the performance states of MTSB reaches the optimum and has almost no failure possibility at the corresponding stage; *d*(*λ*_2_), *d*(*λ*_3_) ∈ [−15%, 0%), which shows that the performance states of MTSB is normal, and the degree failure of OVPS is very small; *d*(*λ*_4_), *d*(*λ*_5_), *d*(*λ*_6_) ∈ [−30%, −15%), which shows that the performance states of MTSB is gradually becoming worse, and the degree failure of OVPS is gradually increasing; *d*(*λ*_7_), *d*(*λ*_8_) < −30%, which shows that the performance states of MTSB is worse, and the degree failure of OVPS is very large. Thus, taking appropriate remedial measures are necessary steps before 5134 min, which can avoid serious safety accidents that are caused by the failure of OVPS.

Figure 22 also shows that the values of PMRR calculated using the second method are smaller than those calculated using this method. The values of PMRR, *d*(*λ*_4_), *d*(*λ*_5_), *d*(*λ*_6_), *d*(*λ*_7_), *d*(*λ*_8_) < −30%, which shows that the degree failure of OVPS is very large after the corresponding time period of the 4th time series. However, this is less in line with the actual degradation process of OVPS of MTSB. Because the interference of random factors during the service process of machine tool spindle bearings (MTSB) was not taken into account.

In summary, the model realizes on-line monitoring of the degradation process of OVPS, which can give timely feedback so as to take preventive and remedial measures before the failure of OVPS for MTSB. Compared to the method in the Reference [8], the vibration threshold does not need to be set manually. Compared to other AI prediction methods, it does not require training on vibration signals, and the adjustment process of parameter is also omitted. In addition, there is no requirement for the length of time series. It is worth noting that, if there is an amount of missing data in the process of signal acquisition, the evaluation results of this approach may be inaccurate, and it cannot diagnose which component of bearings the fault occurred on.

## 4. Conclusions

By means of the Poisson process theory and taking variation probability as a time variable, the proposed model can effectively realize the dynamic evaluation for the degradation process of the OVPS for MTSB. This provides a technical and theoretical basis for on-line health detection and fault diagnosis of spindle bearings.

- The variation probability, obtained using the maximum entropy method and the Poisson counting principle, can accurately describe the degradation information and evolution process of the OVPS of MTSB.
- Considering the interference of random factors, the least-squares method by polynomial fitting, fused into the grey bootstrap maximum entropy method, can be used to calculate the dynamic mean uncertainty, so as to evaluate the random fluctuation state of OVPS.
- The results show the maximum relative errors between the estimated true value and the actual value of the PMR are 6.55% and 9.91% for the MTSB in the two studied cases: Case 1 and Case 2, respectively. Appropriate remedial measures should be taken before 6773min and 5134 min for the MTSB in the two studied cases: Case 1 and Case 2, respectively, which can avoid serious safety accidents caused by the failure of OVPS.

## Figures and Tables

**Table 1:** Performance degradation stage divided.

Data Point	Degradation Stage
1st to 472nd	Initial wear stage
473rd to 2574th	Optimal performance state
2575th to 6659th	Normal wear stage
6660th to 7446th	Degeneration stage
7447th to 7793rd	Deterioration stage

**Table 2:** Fitting using least-square method.

Order Number *q* of Polynomials	Expressions of Polynomials	Correlation Coefficient R^2^
Second order	*G*_2_(*λ_n_*) = 0.0155*λ_n_*^2^ − 0.1162*λ_n_* + 0.2065	0.7863
Third order	*G*_3_(*λ_n_*) = 0.0048*λ_n_*^3^ − 0.0712*λ_n_*^2^ + 0.3181*λ_n_* − 0.3193	0.9390
Fourth order	*G*_4_(*λ_n_*) = 0.0009*λ_n_*^4^ − 0.0162*λ_n_*^3^ + 0.0958*λ_n_*^2^ − 0.1751*λ_n_* + 0.0900	0.9725
Fifth order	*G*_5_(*λ_n_*) = −0.0002*λ_n_*^5^ + 0.0076*λ_n_*^4^ − 0.0900*λ_n_*^3^ + 0.4533*λ_n_*^2^ − 0.9059*λ_n_* + 0.5576	0.9860
Sixth order	*G*_6_(*λ_n_*) = −0.00008*λ_n_*^6^ + 0.0027*λ_n_*^5^ − 0.0335*λ_n_*^4^ + 0.1897*λ_n_*^3^ − 0.4996*λ_n_*^2^ + 0.5821*λ_n_* − 0.2323	0.9955

**Table 3:** Fitting results of polynomials.

Sequence Number of Time Series	Variation Probability			
	Third-Order Polynomial	Fourth-Order Polynomial	Fifth-Order Polynomial	Sixth-Order Polynomial
1	−0.0676	−0.00457	0.0224	0.0090
2	0.0707	0.0078	−0.0462	−0.0033
3	0.1243	0.0615	0.0524	0.0266
4	0.1222	0.1120	0.1477	0.1161
5	0.0933	0.1358	0.1712	0.1833
6	0.0665	0.1303	0.1293	0.1682
7	0.0706	0.1138	0.0764	0.0932
8	0.1346	0.1259	0.0879	0.0664
9	0.2873	0.2268	0.2331	0.2247
10	0.5577	0.4980	0.5486	0.6187
11	0.9746	1.0418	1.0108	1.0379

**Table 4:** Estimated uncertainties *U_λn_* of fitting effect using polynomials.

Sequence Number of Time Series *n*	Estimated Uncertainties *U_λn_*	Sequence Number of Time Series *n*	Estimated Uncertainties *U_λn_*
1	0.2010	7	0.1016
2	0.2361	8	0.1537
3	0.2088	9	0.1478
4	0.0878	10	0.2538
5	0.1935	11	0.1491
6	0.2194		

**Table 5:** Fitting using least-square method (Case 2).

Order Number *q* of Polynomials	Expressions of Polynomials	Correlation Coefficient R^2^
First order	*G*_1_(*λ_n_*) = 0.1567*λ_n_* − 0.3046	0.8905
Second order	*G*_2_(*λ_n_*) = 0.0180*λ_n_*^2^ − 0.0050*λ_n_* − 0.0350	0.9373
Third order	*G*_3_(*λ_n_*) = −0.0025*λ_n_*^3^ + 0.0513*λ_n_*^2^ − 0.1323*λ_n_* + 0.0873	0.9404
Fourth order	*G*_4_(*λ_n_*) = −0.0012*λ_n_*^4^ + 0.0197*λ_n_*^3^ − 0.0829*λ_n_*^2^ + 0.1757*λ_n_* − 0.1222	0.9428
Fifth order	*G*_5_(*λ_n_*) = −0.0021*λ_n_*^5^ + 0.0458*λ_n_*^4^ − 0.3704*λ_n_*^3^ + 1.3750*λ_n_*^2^ − 2.1970*λ_n_* + 1.1590	0.9596
Sixth order	*G*_6_(*λ_n_*) = −0.0021*λ_n_*^6^ + 0.0561*λ_n_*^5^ − 0.5690*λ_n_*^4^ + 2.8454*λ_n_*^3^ − 7.2531*λ_n_*^2^ + 8.8018*λ_n_* − 3.8808	0.9910

**Table 6:** Fitting results of polynomials (Case 2).

Sequence Number *n* of Time Series	Variation Probability					
	First-Order Polynomial	Second-Order Polynomial	Third-Order Polynomial	Fourth-Order Polynomial	Fifth-Order Polynomial	Sixth-Order Polynomial
1	−0.1479	−0.0220	0.0039	−0.0109	0.0103	−0.0017
2	0.0088	0.0269	0.0083	0.0359	−0.0324	0.0263
3	0.1655	0.1118	0.0857	0.0922	0.1433	0.0391
4	0.3222	0.2326	0.2213	0.2024	0.2475	0.3113
5	0.4789	0.3893	0.4003	0.3812	0.3365	0.4069
6	0.6356	0.5820	0.6078	0.6138	0.5626	0.4782
7	0.7923	0.8107	0.8290	0.8556	0.9230	1.0136
8	0.949	1.0752	1.0490	1.0325	1.0089	1.0357

**Table 7:** Estimated uncertainties *U_λn_* of fitting effect using polynomials (Case 2).

Sequence Number *n* of Time Series	Estimated Uncertainties *U_λn_*	Sequence Number *n* of Time Series	Estimated Uncertainties *U_λn_*
1	0.2071	5	0.1751
2	0.1058	6	0.1950
3	0.1605	7	0.2890
4	0.1734	8	0.3041

## Data Availability

The data used to support the findings of this study are available from the corresponding author upon request.

## Nomenclature

*x*(*k*)	*k*th performance data in the intrinsic sequence.
*k*	order number of performance data in intrinsic sequence.
*N*	total number of performance data in the intrinsic sequence.
*x_n_*(*k*)	*k*th performance data of the *n*th time series.
*n*	order number of time series.
*f*(*x*)	probability density function of continuous variable *x*.
ln*f*(*x*)	logarithm of the probability density function *f*(*x*).
*S*	feasible domain of the performance random variable *x*.
*S*_1_	lower-bound value of the feasible domain.
*S*_2_	upper-bound value of the feasible domain.
*i*	order number of origin moment.
*m_i_*	*i*th order origin moment.
*x^i^*	coefficient of the function *f*(*x*).
*c_i_*	(*i* + 1)th Lagrange multiplier.
*a; b*	mapping parameters.
*α*	significant level.
*P*	confidence level.
*N_n_*_1_	Number showing that performance data are less than *X*_L1_ for the *n*th time series.
*N_n_*_2_	Number showing that performance data are more than *X*_U1_ for the *n*th time series.
*G_q_*(*λ*)	*q*th order polynomial.
*q*	order number of polynomial function.
*p_qγ_*	coefficient of the power function *λ^γ^*.
***Y***(*n*)	data sample of variation probability of OVPS for the *n* time series.
*y_n_*(*u*)	*u*th data in the variation probability data sample for the *n* time series.
***V*** *_β_*	*β*th bootstrap re-sampling sample.
*B*	times of the bootstrap re-sampling.
*v**_β_*(Θ)	Θth data in the *β*th bootstrap re-sampling sample.
*u*	time variable.
*c*_1_; *c*_2_	coefficients to be estimated.
*λ_n_*_L_; *λ_n_*_U_	lower-bound value and upper-bound value of the variation probability data sample for the *n*th time series.
*U_λn_*	estimated uncertainty of variation probability.
*P_R_*	reliability of the polynomials fitting effect using least-squares method.
*U*_mean_	dynamic average uncertainty.
*|_PR_*_ = 100%_	calculation process is under the condition of *P_R_* = 100%.
*e*	number of occurring failure events.
*Q*	probability of failure events occurring *e* times.
*R*(*λ_n_*)	function of the variation probability *λ_n_*.
*R*(*θ*_1_)	PMR for the intrinsic series of MTSB.
*R*(*θ_n_*)	PMR for the *n*th time series of MTSB.
*d*(*θ_n_*)	PMRR for the *n*th time series of MTSB.
MTSB	machine tool spindle bearings.
VPMR	vibration performance maintaining reliability.
OPS	optimal performance state.
ULBC	upper- and lower-bound curves.
SPSB	Super-precision spindle bearings.
PMR	Performance maintaining reliability.
PMRR	performance maintaining relative reliability.
